# UCHL5 promotes hepatocellular carcinoma progression by promoting glycolysis through activating Wnt/β-catenin pathway

**DOI:** 10.1186/s12885-023-11317-z

**Published:** 2024-05-21

**Authors:** Baishun Wan, Ming Cheng, Tao He, Ling Zhang

**Affiliations:** Department of Hepatobiliary and Pancreatic Surgery, The Affiliated Cancer Hospital of Zhengzhou University, No. 127, Dongming Road, Zhengzhou, Henan Province 450008 China

**Keywords:** Hepatocellular carcinoma, *UCHL5*, Wnt/β-catenin pathway, Glycolysis, Ubiquitination

## Abstract

**Background:**

Hepatocellular carcinoma (HCC) is highly malignant with a dismal prognosis, although the available therapies are insufficient. No efficient ubiquitinase has been identified as a therapeutic target for HCC despite the complicating role that of proteins ubiquitination plays in the malignant development of HCC.

**Methods:**

The expression of ubiquitin carboxyl terminal hydrolase L5 (*UCHL5*) in HCC tumor tissue and adjacent normal tissue was determined using the cancer genome atlas (TCGA) database and was validated using real-time quantitative polymerase chain reaction (RT-qRCR), Western blot and immunohistochemistry (IHC), and the relation of *UCHL5* with patient clinical prognosis was explored. The expression of *UCHL5* was knocked down and validated, and the effect of *UCHL5* on the biological course of HCC was explored using cellular assays. To clarify the molecular mechanism of action of *UCHL5* affecting HCC, expression studies of Adenosine triphosphate adenosine triphosphate (ATP), extracellular acidification (ECAR), and glycolysis-related enzymes were performed. The effects of *UCHL5* on β-catenin ubiquitination and Wnt signaling pathways were explored in depth and validated using cellular functionalities. Validation was also performed in vivo.

**Results:**

In the course of this investigation, we discovered that *UCHL5* was strongly expressed in HCC at both cellular and tissue levels. The prognosis of patients with high *UCHL5* expression is considerably worse than that of those with low *UCHL5* expression. *UCHL5* has been shown to increase the degree of glycolysis in HCC cells with the impact of stimulating the proliferation and metastasis of HCC cells in both in vivo and in vitro. *UCHL5* downregulates its degree of ubiquitination by binding to β-catenin, which activates the Wnt/β-catenin pathway and accelerates HCC cell glycolysis. Thereby promoting the growth of the HCC.

**Conclusions:**

In summary, we have demonstrated for the first time that *UCHL5* is a target of HCC and promotes the progression of hepatocellular carcinoma by promoting glycolysis through the activation of the Wnt/β-catenin pathway. *UCHL5* may thus serve as a novel prognostic marker and therapeutic target for the treatment of HCC.

**Supplementary Information:**

The online version contains supplementary material available at 10.1186/s12885-023-11317-z.

## Introduction

The second most prevalent cause of mortality worldwide and the sixth most common malignancy is hepatocellular carcinoma (HCC) [1, 2]. Hepatocellular carcinoma, which has one of the highest rates of occurrence in the world, especially in China, is a serious threat to human health and life expectancy [3]. The American Association for the Study of Liver Disease’s recommendations call for surgical resection (SR) and radiofrequency ablation (RFA) for early HCC. Patients who got RFA, however, exhibited worse long-term survival than those who received SR. For early-stage HCC, surgical excision is still regarded as the best course of action [4]. The majority of HCC patients are in advanced stages at the time of diagnosis and cannot receive surgical treatment because there are no distinct clinical symptoms. The conventional therapy for intermediate-stage, unresectable hepatocellular carcinoma is transarterial chemoembolization (TACE). A potential method called drug-eluting beads (DEB)-TACE is anticipated to increase the effectiveness and safety of traditional TACE [5]. Targeted therapy is one of the main treatment methods for advanced liver cancer [6], Sorafenib is the only drug approved by the FDA for targeted therapy of advanced hepatocellular carcinoma, but it will inevitably appear as primary or secondary drug resistance [7]. Immune checkpoint inhibitor seems to be a promising new direction, but only some patients with hepatocellular carcinoma are effective for Immunocheckpoint inhibitor [8]. Therefore, the creation of novel therapeutic targets and individualized precision medicine for hepatocellular carcinoma is urgently required.

The liver itself is an important metabolic organ of the human body. Metabolic abnormalities in hepatocellular carcinoma appear to be particularly prominent in comparison to other solid tumors. Tumor reprogramming by Metabolic is one of the important malignant hallmarks of cancer progression, which can meet the energetic demand of unlimited cancer cell proliferation by increasing glucose uptake [9]. Many of the molecules involved in the multistep process of glucose metabolism have been linked to both HCC formation and progression as well as being unfavourable prognostic indicators of disease, including GLUT1 [10], PKM2 [11], and *HK2* [12] and so on. Aberrant expression of these key factors in HCC provides sufficient ATP to HCC cells via the “Warburg effect”. [13].

In addition to the abnormal protein expression caused by abnormal transcription, another mechanism of abnormal protein expression in tumors is the disorder of the protein degradation pathway. The most common is the ubiquitination degradation pathway. By reversibly attaching to the component of the 26 S proteasome, ubiquitin C-terminal hydrolase L5 (*UCHL5*) / *UCH37* is a cysteine protease of the ubiquitin C-terminal hydrolase (UCHs) family, Ub can be removed from the distal end of multi Ub chain, resulting in reduced degradation of the deubiquitinated target protein and increased expression in cells [14–17]. However, *UCHL5* has also been observed to specifically promote the destruction of inducible nitric oxide synthase and *IκB-α* on the proteasome [18]. *UCHL5* is abnormally expressed in a variety of tumors, but its role in tumors is not completely consistent. In gastric cancer [19] and lymph node-positive renal cancer [20], However, in cases of lung adenocarcinoma [21] and ovarian cancer [22], its high expression is not linked to a positive prognosis, Its high expression is linked to a bad prognosis, demonstrating the complexity of *UCHL5’s* function in malignancies. Previous research on HCC has discovered that the expression of *UCHL5* is elevated and encourages the spread of HCC cells [23], but its specific mechanism has not been completely clear, and further research is required to clarify its specific role and mechanism.

The aim of the present investigation was to investigate *UCHL5’s* function in HCC. The findings demonstrated that *UCHL5* expression in HCC was substantially greater than in nearby tissues. Patients with high *UCHL5* expression have a far poorer prognosis than those with low *UCHL5* expression. In vivo and in vitro, *UCHL5* encourages the growth and metastasis of HCC cells. This impact is accomplished by encouraging the cells’ glycolysis. *UCHL5* reduces its amount of ubiquitination by binding to β-catenin, which in turn activates the Wnt/β-catenin pathway, resulting in accelerated HCC development. These findings offer a fresh window into the occurrence and progression of HCC and offer a foundation for therapeutic care.

## Materials and methods

### Cell lines and cell culture

The Cell Resource Center of the Institute of Basic Medicine, Chinese Academy of Medical Sciences contributed a normal liver cell line (HL-7702) and HCC cell lines (BEL7402, PLC/PRF/5, Huh7, HepG2, HepG3B, and SNU449) (Beijing, China). The cell lines were grown in a 37 °C cell incubator with 5% CO2 using Roswell Park Memorial Institute 1640 (RPMI-1640) media from Glibco in Massachusetts, USA, supplemented with 10% fetal bovine serum (FBS) from HyClone in South Logan, UT, USA, 100 g/mL streptomycin, and 100 U/mL penicillin.

### shRNA transfection and construction of stable knocked-down cell line

ViewSolid Biotech sold *UCHL5* shRNAs and negative control shRNA (Beijing, China). The *UCHL5* shRNA sequences were as follows: sh-RNA-1 *UCHL5*: GCAAAGAAAGCUCAGGAAATT, sh-RNA-2: 5’-GAUCAAGGUAAUAGUAUGUTT-3’, Control: 5’-UUCUCCGAACGUGUCACGUTT-3’. According to the manufacturer’s instructions, all of the aforementioned siRNAs were transfected into cells using jetPRIME reagent (Polyplus). Negative control and *UCHL5*-shRNA lentiviral particles were bought from Genechem to create a cell line that would permanently silence the *UCHL5* gene (Shanghai, China). As directed by the manufacturer, cells were transfected with sh-Control or *UCHL5*-shRNA lentiviral particles. To choose stably transfected cells, puromycin (2 µg/mL, cat. no. P7130; Sigma-Aldrich; Merck Millipore) was utilized. The effectiveness of *UCHL5*’s knockdown was assessed by Western blotting analysis.

### RNA isolation and quantitative real-time PCR (RT-qRCR)

Trizol reagent was used to extract total RNA (Invitrogen, Carlsbad, CA, USA). Then, using a NanoDrop ND-100 spectrophotometer, the absorbance at 260 nm was used to calculate the amount of RNA (NanoDrop Technologies, Rockland, DE, USA). Using the PrimeScript RT Reagent Kit, the RNA was reverse transcribed into complementary deoxyribose nucleic acid (cDNA) (Takara Bio, Kusatsu, Japan). SYBR Premix Ex Taq II (TaKaRa) was used for quantitative real-time PCR, which was conducted utilizing Applied Biosystems® 7500 Real-Time PCR Systems (Thermo Fisher Scientific, Waltham, MA, USA). Using the 2-ΔΔCt technique, the relative expression relative to internal parameters (GAPDH) was determined. The following PCR primers were utilized: *UCHL5*, forward: 5’GAGTGGTGCCTCATGGAAAG3’; reverse: 5’ CAAGTCGGGAGTCCTGAACC3’; GLUT1, forward: 5’GGCCAAGAGTGTGCTAAAGAA3’; reverse: 5’ACAGCGTTGATGCCAGACAG3’; *HK2*, forward: 5’GAGCCACCACTCACCCTACT3’; reverse: 5’ CCAGGCATTCGGCAATGTG3’; PKM2, forward: 5’ATGTCGAAGCCCCATAGTGAA3’; reverse: 5’TGGGTGGTGAATCAATGTCCA3’; *LDHA*, forward: 5’ATGGCAACTCTAAAGGATCAGC3’; reverse: 5’CCAACCCCAACAACTGTAATCT3’; *LDHB*, forward: 5’TGGTATGGCGTGTGCTATCAG3’; reverse: 5’TTGGCGGTCACAGAATAATCTTT3’; *PDK1*, forward: 5’CTGTGATACGGATCAGAAACCG3’; reverse: 5’TCCACCAAACAATAAAGAGTGCT.

### Cell counting kit-8 (CCK-8) assay

The treated cells were plated in 96-well plates (5 × 10^3^ cells/well) in 200 µL culture media in accordance with the experimental conditions. According to the manufacturer’s instructions, the CCK-8 assay (Dojindo, Kumamoto, Japan) was carried out.

### Transwell assay

Transwell assays were carried out using transwell chambers on cells transfected for 24 h with sh-Control or *UCHL5*-shRNA in accordance with the experimental conditions (Corning Life Science, MA, USA). The cells were added to the upper chamber for the migration experiments at a density of 4 × 10^4^ cells per 200 µL in RPMI-1640 media without FBS, and they were incubated there at 37 °C and 5% CO_2_. Matrigel (Corning, Corning, NY, USA) was pre-plated in the bottom of the top chamber of the Transwell chamber (50 µL) for invasion experiments and then incubated at 37 °C for 30 min. Cells that were still on the lower side of the filter were incubated for another 24–48 h before being fixed with ethanol, stained with Reiter coloring for 1 min, and then redyed with mixed Giemsa for 1 h. Using an Olympus microscope from Tokyo, Japan, the stained cells were counted and captured on camera at a magnification of about 200. At least five fields were tallied and statistically examined at random.

### Colony formation experiment

The transfected HCC cells were seeded onto 12-well plates at a density of 300 cells per well, and they were subsequently cultured for two weeks at 5% CO_2_ and 37 °C. The generated cell colonies were stained for five minutes with 0.1% crystal violet after being fixed with 4% paraformaldehyde. Under a microscope, the number of colonies was counted and inspected.

### Clinical samples

From January 2016 to December 2021, a total of 76 pairs of HCC tissues and matched normal liver tissues were collected from the Affiliated Cancer Hospital of Zhengzhou University Hospital. The patient had no operation, radiotherapy, or chemotherapy before the operation, and all patients knew and agreed. Ethical approval for this study was obtained from the ethics committee of the Affiliated Cancer Hospital of Zhengzhou University Hospital.

### Western blot and immunoprecipitation

Radioimmunoprecipitation assay (RIPA) lysate that had been previously refrigerated was used to lyse the transfected cells or tissue. The BCA technique was used to measure the concentration of total protein (Beyotime, China). SDS-PAGE was used to separate the protein lysates and the membrane was subsequently coated with PVDF. The primary antibody was incubated with the PVDF membrane at 4 °C overnight as per the manufacturer’s instructions. The PVDF membrane was then incubated for 2 h at room temperature with a goat anti-rabbit secondary antibody that was HRP-labeled (1:5000, CAS #pr30011, Proteintech, China), after which the bands were identified using a Tianneng Tanon-5200 automated chemiluminescence image analysis equipment (China). Cell lysates were combined with antibody and protein G-sepharose beads (Cell Signaling Technology) for immunoprecipitation at 4 °C for at least 6 h. The immunoprecipitated proteins were then heated at 95 °C for 5 min while being treated with 2X sampling buffer. The blots were cut prior to hybridisation with antibodies during blotting.

### Wound healing assays

Assays for the healing of scratch wounds were conducted 48 h after transfection. When confluence reached around 100%, the cells were planted on six-well plates and injured by making a straight scratch with a 200 µL pipette tip. Images of migration were taken at zero hours after scratching. The ImageJ program was used to evaluate and quantify the blank space. Three times each of the tests were run.

### Animal experiments

After being acclimated for a week, ten BALB/c female nude mice (4–6 weeks old, 18-20 g) were obtained from Beijing Vital River Laboratory Animal Technology Co, Ltd. Mice were subcutaneously implanted with the cells that were suspended in 200 µl of PBS (1 × 10^7^ stable shUCHL5). The weight of the animals and the tumors’ long diameter and short meridian were frequently checked after cell injection, which took place around a week later. All mice were sacrificed via cervical dislocation under anesthesia using isoflurane. These mice were euthanized after 5 weeks in accordance with the criteria set by the Zhengzhou University Committee on Animal Care, and the weights of the tumors were measured, along with the tumor tissues being removed aseptically. 2 × 10^6^ cells were injected into the caudal vein of mice in order to identify cell metastasis. Daily observations of the mice’s health were made, and after four weeks of feeding, the animals were put to death via cervical dislocation. The lungs were then removed, and HE staining was carried out. The Zhengzhou University Institutional Review Board gave its approval to animal testing.

### Kyoto Encyclopedia of Genes and Genomes (KEGG) pathway enrichment analysis of *UCHL5* co-expressed genes in The Cancer Genome Atlas (TCGA)

It is necessary to download the raw data files of TCGA data (https://portal.gdc.cancer.gov), which contain expression matrices, annotation files and related metadata of all samples. KEGG enrichment analysis was performed on 100 correlated genes, with the parameter setting: *p* < 0.05. The enrichment analysis was performed by the DAVID database (https://david.ncifcrf.gov/home.jsp).

### Immunohistochemistry (IHC) staining

Purchased from Maixin Biotechnology were the reagents (Fuzhou, China). Execute particular experimental procedures in accordance with the guidelines. According to the staining intensity, IHC grading was done (0, negative; 1, weak; 2, moderated; 3, strong).

### Protein identification by mass spectrometry

The gel was stained with silver as per the instructions, and the isolated peptides were examined using nano-LC-MS/MS (AB SCIEX TripleT OF 5600, USA) as per the instructions.

### Lactate production, glucose uptake and ATP levels measurement

Hepatocellular carcinoma cells are handled in accordance with the needs of the experiment. Using a Lactate Colorimetric Assay kit II, the buildup of lactate in the medium was found (K627-100, BioVision, USA). Utilizing a glucose colorimetric assay kit, the quantity of glucose was determined (K606-100, Bio Vision, USA). The ENLITEN® ATP Assay System (Promega) was used to quantify ATP release in order to examine the ATP in supernatants.

### Extracellular acidification rate (ECAR) analysis

Indicated cells were plated onto Seahorse XFe24 Cell Culture Microplates for the ECAR assay (Agilent, Palo Alto, CA, USA). According to the manufacturer’s instructions, the test was carried out using the Seahorse XF Glycolysis Stress Test Kit (103020-100; Agilent). The XFe24 wave program was used to gather and analyze the data (Agilent).

### Luciferase assay

HCC cells were transfected with TOPflash, a luciferase reporter plasmid with Wnt luciferase activity, or control FOPflash. TOPflash encodes the three primary untranslated regions (3′-UTR) of *UCHL5*, whereas FOPflash is an empty vector. The Dual-Luciferase Reporter Assay System (Promega, Madison, WI) was used to quantify luciferase activities in accordance with the manufacturer’s instructions.

### Statistical analysis

GraphPad Prism was used to perform all statistical analyses. Three different experiments’ means and standard deviations (SD) were used to illustrate the results of the cell culture experiment. The Student’s t-tests were used to evaluate the differences between the two groups. The threshold for statistical significance was P < 0.05.

## Results

### Hepatocellular carcinoma patients who have high levels of *UCHL5* expression have a bad prognosis

**Fig. 1:**
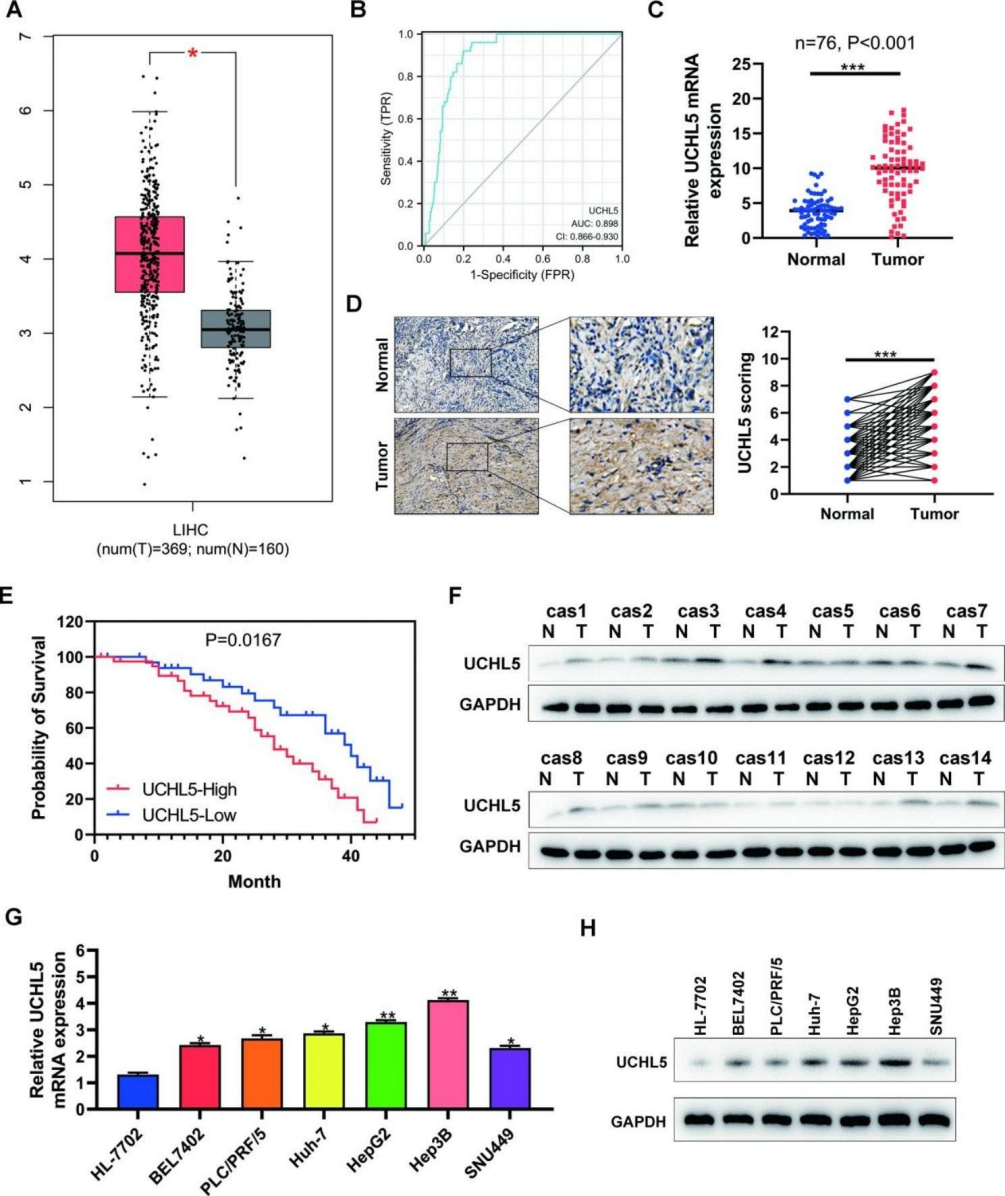
***UCHL5*is highly expressed in hepatocellular carcinoma, and the prognosis of patients with high expression is poor**. (**A**) Expression of the *UCHL5* was validated in 369 HCC tissues and 160 normal tissues with GEPIA. Tumor tissue is shown in red, and normal tissue is shown in gray; (**B**) The AUC value of a single *UCHL5* gene in hepatocellular carcinoma; (**C**, **D**) The mRNA and protein expression of *UCHL5* in hepatocellular carcinoma and adjacent normal tissues were analyzed by using RT-qPCR and immunohistochemistry; (**E**) Prognosis of patients with different *UCHL5* expression levels; (**F**) The expression of *UCHL5* in 14 cases of hepatocellular carcinoma and adjacent tissues was detected by Western blot; (**G**, **H**) The expression of *UCHL5* mRNA and protein expression in human normal hepatocytes (HL-7702) and six hepatocellular carcinoma cells (BEL7402, PLC/PRF/5, Huh-7, HepG2, HepG3B and SNU449). Data represent the mean ± SD of three independent experiments. GAPDH as internal parameter. (*p < 0.05, **p < 0.01 and ***p < 0.001)

We initially used GEPIA online TCGA-LIHC data to identify the expression level of *UCHL5* in HCC and surrounding tissues in order to investigate the function of *UCHL5* in HCC (Fig. 1A), the expression of *UCHL5* in HCC was significantly increased, and the accuracy of 1-year prognosis prediction was high. The AUC value of a single *UCHL5* gene was 0.898 (Fig. 1B). We further confirmed the *UCHL5* expression differentially in fresh tissues between HCC and normal tissues. In HCC tissues, *UCHL5* mRNA was highly up-regulated (Fig. 1C), and the protein level followed a similar pattern (Fig. 1D F and S1A). We utilized the Kaplan Meier plotter to assess the prognostic differences of HCC patients with varying levels of *UCHL5* expression in order to understand the function of *UCHL5* expression in the prognosis of HCC. The outcomes demonstrated that patients with high *UCHL5* expression in their HCC had a bad prognosis (Fig. 1E). Then, we discovered that hepatocytes and other HCC cells expressed *UCHL5* mRNA and protein. The findings demonstrated that hepatoma cells expressed more *UCHL5* mRNA and protein than normal hepatocytes did and the most significant differential expression of *UCHL5* was observed in HepG2 and Hep3B cell lines (Fig. 1G H and S1B). Therefore, HepG2 and Hep3B cell lines were selected as target cell lines for subsequent cell experiments.

### *UCHL5* promotes the growth and metastasis of HCC cells in vitro

**Fig. 2:**
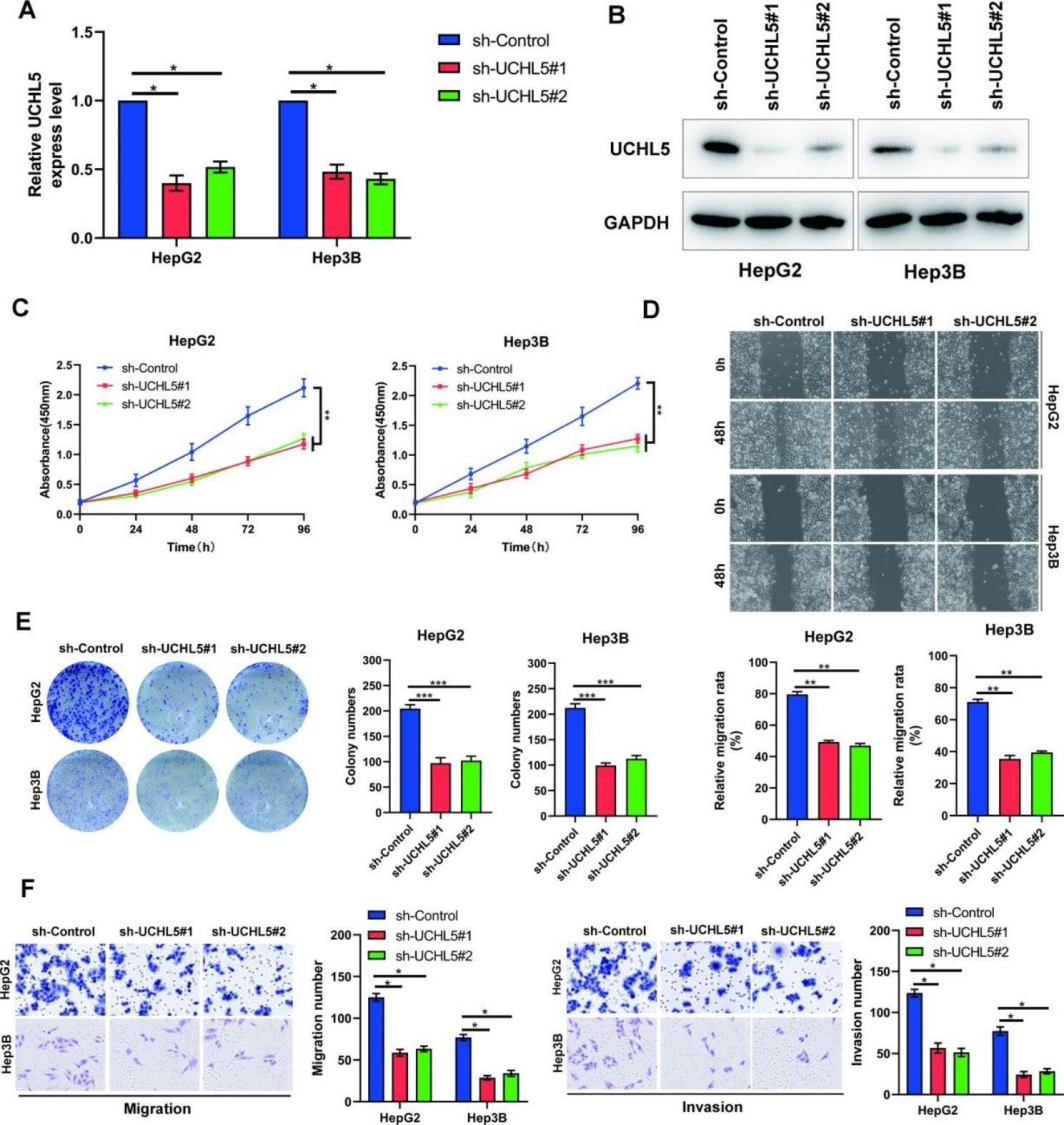
***UCHL5*promotes the proliferation and metastasis of HCC cells in vitro**. (**A**, **B**) ShUCHL5 or sh-control were transfected into HepG2 and Hep3B cells. The mRNA and protein levels of *UCHL5* were examined by RT-qPCR and western blot; (**C**, **E**) The effect of UCHL5 on HCC cell proliferation was determined by CCK-8 (**C**) and colony formation (**E**); (**D**) Cell scratch assay to determine the effect of *UCHL5* on the migratory ability of HCC cells; (**F**) The effect of *UCHL5* on migration (left) and invasion (right) were evaluated by Transwell assays. Data represent the mean ± SD of three independent experiments. GAPDH as internal parameter. (*p < 0.05, ** *p* < 0.01 and *** *p* < 0.001)

The function of *UCHL5* in HCC cells was then investigated. In the beginning, we reduced *UCHL5* expression in HCC cells. At the mRNA and protein levels, respectively, Fig. 2A, B and S1C show the knockdown effectiveness. The capacity of cells to proliferate changed when *UCHL5* expression in cells was altered, and CCK-8 was utilized to identify this shift. The findings demonstrated that *UCHL5* knockdown greatly reduced HCC cell growth and that this effect was time-dependent (Fig. 2C), and the colony experiment confirmed that the same trend was obtained in long-term proliferation (Fig. 2E). Further, explore whether *UCHL5* affects HCC cell metastasis. The results show that the knockdown of *UCHL5* significantly inhibited HCC cell metastasis. The Wound healing test (Fig. 2D) and Transwell migration and invasion test (Fig. 2F) both supported this finding. In HCC cells, *UCHL5* knockdown dramatically reduced the capacity for cell metastasis. These findings demonstrate that *UCHL5* promotes the in vitro proliferation and metastasis of HCC cells.

### UCHL5 promotes glycolysis of HCC cells

**Fig. 3:**
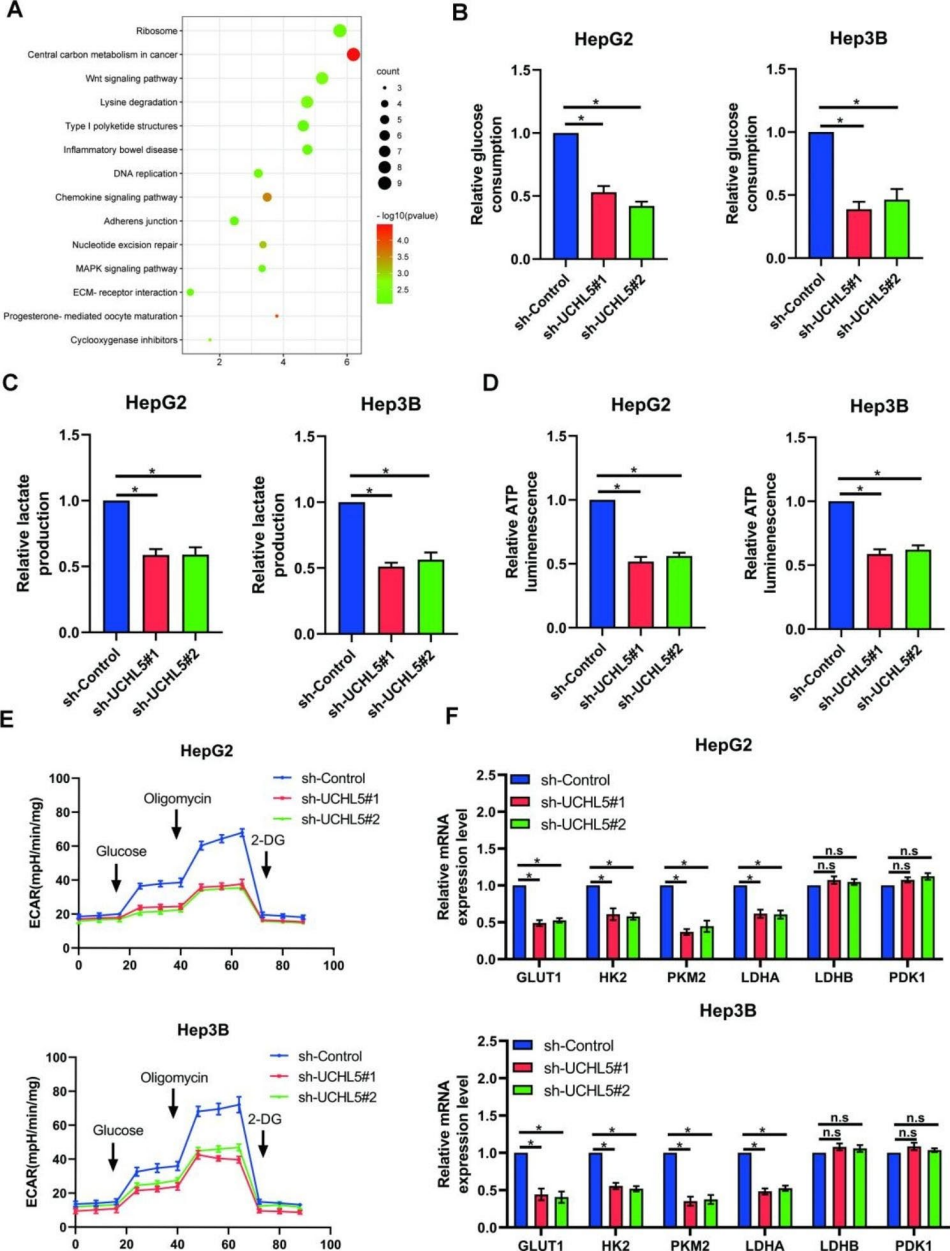
***UCHL5*promotes the glycolysis of HCC cells**. (**A**) UCHL5 co-expression genes in TCGA were enriched in KEGG pathways; (**B**, **C**, **D**) Detection of glucose consumption (**B**), lactate production (**C**), intracellular ATP level (**D**) and ECAR(**E**) in transfected cells; (**F**) The mRNA levels of GLUT1, PKM2, *HK2*, *LDHA*, *LDHB*, and *PDK1* were examined by RT-qPCR after transfected with shUCHL5 or sh-control. Data represent the mean ± SD of three independent experiments. (*p < 0.05)

We examined the pathway enrichment of *UCHL5* in HCC in order to better understand how *UCHL5* encourages the growth and metastasis of HCC cells [24–26]. The findings demonstrated that the glycolysis route underwent the greatest multiple of change (Fig. 3A), which led us to speculate whether *UCHL5* promoted tumor progression by regulating the glycolysis process. In order to verify this conjecture, we measured and detected the change in glucose uptake after the knockdown of *UCHL5* in HCC cells using fluorescently labeled glucose 2-NBDG. The results showed that glucose uptake decreased significantly after the knockdown of *UCHL5* (Fig. 3B). In addition, the intracellular L-lactic acid concentration and ATP concentration decreased significantly in the knockdown *UCHL5* group (Fig. 3C and D). These findings imply that *UCHL5* promotes tumor cell glycolysis, and the drop in intracellular metabolites and ATP following *UCHL5* knockdown supports this hypothesis. In addition, we indirectly reflected the glycolytic ability under different expression states of *UCHL5* by detecting the intracellular acid production ability. The results also found that *UCHL5* significantly improved the glycolytic ability of tumor cells (Fig. 3E). Furthermore, we detected metabolism-related target markers, including GLUT1, PKM2, *HK2*, *LDHA*, *LDHB*, and *PDK1*. Results from RT-qRCR revealed that UCHL5 expression significantly reduced the expression of the target genes GLUT1, PKM2, *HK2*, and *LDHA*, but did not affect the expression of *LDHB* and *PDK1* (Fig. 3F). These findings imply that the high expression of *UCHL5* in tumors stimulates the expression of genes involved in metabolism, allowing for significant glycolysis and increased energy generation to sustain tumor development.

### UCHL5 reduces β-catenin degradation through deubiquitination

**Fig. 4:**
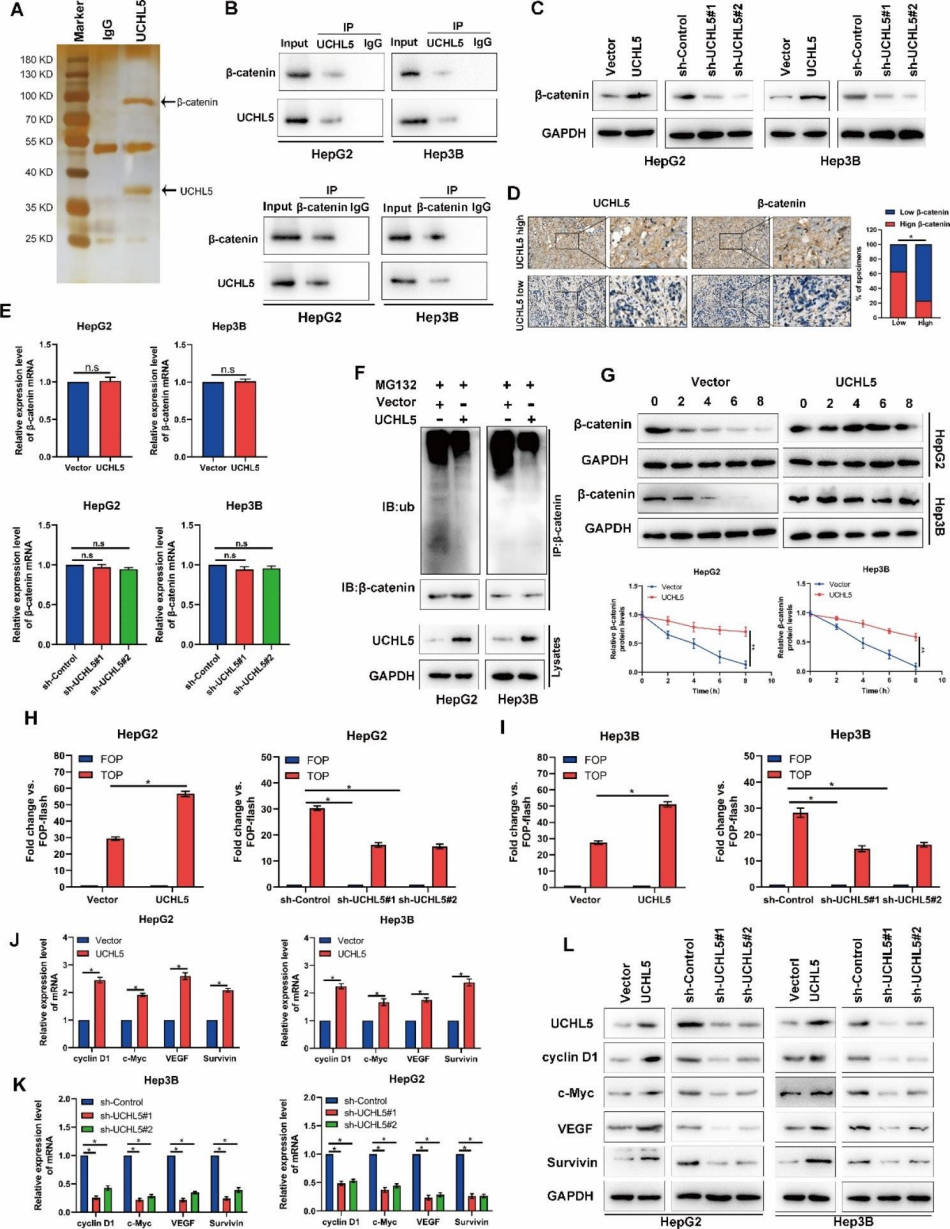
***UCHL5*reduces β-catenin degradation through deubiquitination**. (**A**) Immunoprecipitation of *UCHL5* or IgG was carried out in HCC cells. The precipitated protein was analyzed by SDS-PAGE, silver staining was carried out, and the differential bands were identified by mass spectrometry (MS); (**B**) Co-IP demonstrated the binding between *UCHL5* and β-catenin protein; (**C**, **E**) The mRNA and protein levels of β-catenin were examined by RT-qPCR (**E**) and western blot (**C**) after knockdown or overexpression of *UCHL5*; (**D**) The expression level of β-catenin in different groups (*UCHL5* high or *UCHL5* low) were showed by IHC; (**F**) The protein levels of β-catenin and the ubiquitination level of β-catenin were examined by western blot after overexpression of *UCHL5* or addition of MG132; (**G**) The protein levels of β-catenin were examined in different time points after adding CHX by western blot and gel blots quantification; (**H**, **I**) The TOP/FOP fold change after knockdown or overexpression of *UCHL5*; (**J**, **K**, **L**) The mRNA and protein levels of cyclinD1, c-Myc, VEGF and survivin were examined by RT-qPCR (**J**, **K**) and western blot (**L**) after knockdown or overexpression of *UCHL5*. Data represent the mean ± SD of three independent experiments. GAPDH as internal parameter. (*p < 0.05)

We screened the proteins binding to *UCHL5* by mass spectrometry in HCC cells, it was found that β-catenin is a potential binding protein of *UCHL5* (Fig. 4A). We further conducted a Co-IP experiment to evaluate whether *UCHL5* binds to β-catenin. *UCHL5* binds to β-catenin in HCC cells (Fig. 4B and S1D). Cells’ expression of the β-catenin protein was up-regulated or down-regulated when *UCHL5* was knocked down or overexpressed (Fig. 4C and S1E). IHC further showed that *UCHL5* was positively linked with β-catenin expression in HCC tissues (Fig. 4D), although this expression shift was not brought on by a change in the β-catenin mRNA that *UCHL5* regulates. β-catenin mRNA did not significantly alter following *UCHL5* overexpression or knockdown (Fig. 4E). It showed that the alteration in *UCHL5* expression had no impact on the alteration in mRNA levels for β-catenin. We wonder if *UCHL5*, a protein that is typically deubiquitinated, is involved in the ubiquitination modification of β-catenin so that it can take part in the breakdown process. As a result, we measured the ubiquitination level of the β-catenin protein after overexpressing *UCHL5*. It was found that this resulted in a significant decrease in the ubiquitination of β-catenin (Fig. 4F and S1F). The fact that β-catenin did not substantially decrease with the addition of the protein synthesis inhibitor CHX in the *UCHL5* overexpression group (Fig. 4G) shows that β-catenin protein was more rapidly destroyed in cells without *UCHL5*. Following the alteration of *UCHL5* in cells, the TOP/FOP-flash approach was used to further determine the activity of the Wnt/β-catenin signaling pathway. The findings demonstrated that *UCHL5* dramatically increased the Wnt/β-catenin signaling pathway’s activity (Fig. 4H and I). Additionally, *UCHL5* promotes the mRNA and protein expression levels of CyclinD1, c-Myc, VEGF and Survivin (Fig. 4J, K, L and S1G). These findings imply that *UCHL5* promotes β-catenin ‘s deubiquitination by binding with β-catenin, preventing β-catenin protein from being degraded by ubiquitination, increasing the amount of β-catenin protein in tumor cells, and activating the Wnt/β-catenin signaling pathway, which in turn accelerates the development of HCC.

### *UCHL5* promotes HCC progression through β-catenin

**Fig. 5:**
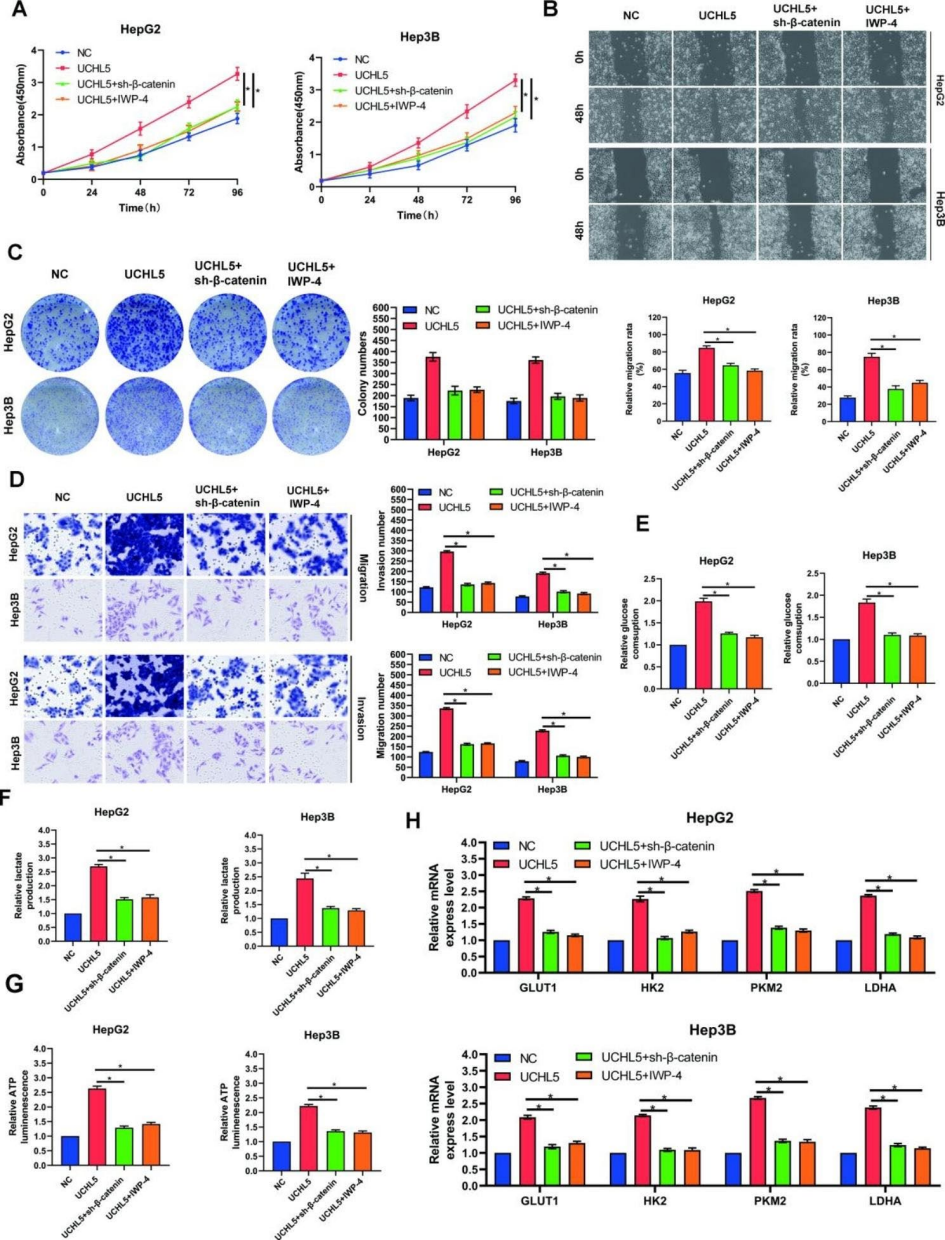
***UCHL5*promotes HCC progression through β-catenin. After changing the expression of*UCHL5***, **β-catenin or adding IWP-4**. (**A**, **C**) The change of HCC cell proliferation was determined by CCK-8 (**A**) and colony formation (**C**) in different time points; (**B**) Cell scratch assay to determine the migration ability of HCC cells; (**D**) The change of migration and invasion ability was evaluated byTranswell assays; (**E**, **F**, **G**) Detection of glucose consumption (**E**), lactate production (**F**) and intracellular ATP level by RT-qPCR (**G**); The mRNA levels of GLUT1, PKM2, *HK2*, and *LDHA* were examined by RT-qPCR. Data represent the mean ± SD of three independent experiments. (*p < 0.05)

We overexpressed *UCHL5* while inhibiting β-catenin or adding a β-catenin inhibitor to further determine if *UCHL5* accelerates HCC development by stimulating the Wnt/β-catenin pathway. The findings demonstrated that the proliferation stimulation brought on by the overexpression of *UCHL5* could be reversed by either knocking down β-catenin or adding a β-catenin inhibitor (Fig. 5A), the same conclusion can be obtained in the long-term proliferation of the set (Fig. 5C). Similar patterns were also discovered in trials involving cell scratching, Tranwell invasion and migration assays. The ability of *UCHL5* to promote metastasis can be increased by using β-catenin inhibitors or decreased by using β-catenin (Fig. 5B and D). Our previous results showed that *UCHL5* promoted the glycolysis of HCC cells. Therefore, we further detected the changes in glucose uptake and lactic acid content in HCC cells after the knockdown of β-catenin or the addition of inhibitors after overexpression of *UCHL5*. The results showed that overexpression of *UCHL5* could promote glucose uptake and lactic acid production in cells, that is, promote glycolysis in cells (Fig. 5E, F). However, its content drastically dropped once β-catenin was knocked down or an inhibitor was added, and it essentially reverted to the level of the control group. Similar to this, ATP produced by cells follows the same trend (Fig. 5G). The mRNA levels of *UCHL5’s* primary metabolic target genes, GLUT1, PKM2, *HK2*, and *LDHA*, were up-regulated after *UCHL5* was overexpressed, and they were almost down-regulated to the control group after β-catenin was knocked down or inhibitors were added (Fig. 5H). These findings demonstrate that the promotion of intracellular glycolysis, which is accomplished by activating the intracellular Wnt/β-catenin pathway in cells to activate the transcription of metabolic-related genes, is responsible for the proliferation and metastasis of *UCHL5* on HCC cells.

### *UCHL5* promotes the proliferation and metastasis of HCC cells in vivo

**Fig. 6:**
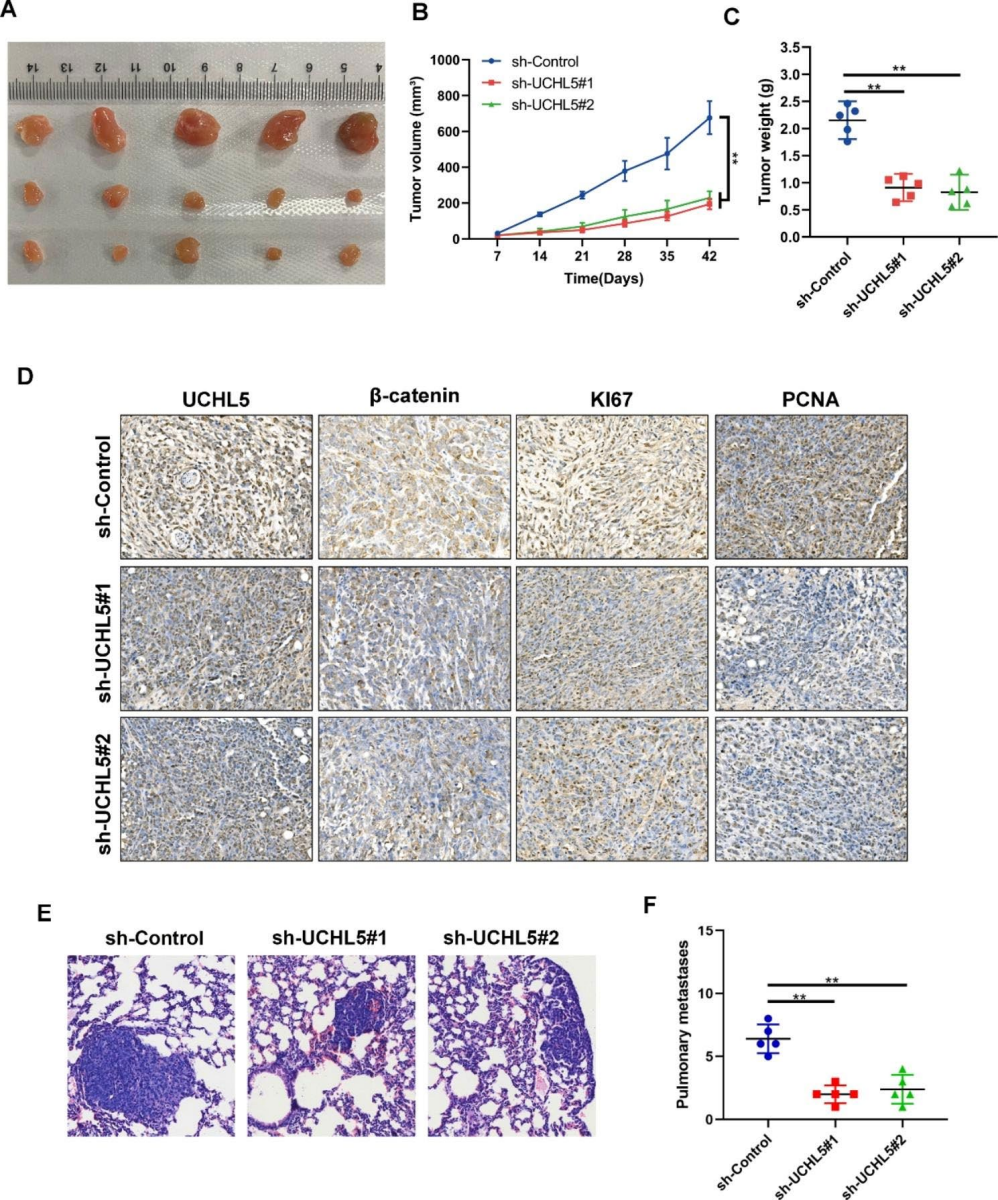
***UCHL5*promotes the proliferation and metastasis of HCC cells in vivo**. (**A**) Images of xenograft tumors derived from transfected cells in nude mice; (**B**) Tumor volume curves were summarized in the line chart. The average tumor volume was expressed as the mean standard deviation of 5 mice; (**C**) The tumor weight was assessed in different groups; (**D**) The expression level of *UCHL5*, β-catenin, KI67, and PCNA in different groups were shown by IHC. (**E**) Representative images of the tail vein-injected mouse models. (**F**) The lung foci number was evaluated. Data represent the mean ± SD of three independent experiments. (**p < 0.01)

We employed the xenograft model to validate subcutaneous carcinogenesis in vivo in order to further support the effect of *UCHL5* on HCC. The capacity of tumor cells to proliferate dramatically diminished in comparison to the control group when *UCHL5* was knocked down (Fig. 6A). After the knockdown of *UCHL5* expression, the volume and weight of tumors were significantly lower than the control group (Fig. 6B C). Immunohistochemical results showed that knockdown of *UCHL5* expression was followed by a significant decrease in the expression of β-catenin, and the expression of proliferation-related markers KI67 and PCNA (Fig. 6D). Lung metastasis analysis showed that down-regulation of UCHL5 expression significantly reduced the diameter and number of metastatic foci in lung tissue (Fig. 6E F). These outcomes demonstrated that *UCHL5* can indeed promote tumor development in vivo.

## Discussion

Most protein breakdown in all eukaryotic cells depends on the ubiquitin-proteasome system (UPS) pathway [27]. The degraded protein is specifically labeled by ubiquitin (Ub), and the proteasome accurately recognizes and degrades the labeled protein [28]. This ubiquitin labeling process depends on ubiquitinase. Of course, this process is reversible. Ubiquitinase prolongs the Ub chain and deubiquitinase (DUBs) mediates cutting multi Ub chain to reversibly regulate [28, 29]. Previous research has demonstrated that both ubiquitinates and deubiquitinases are crucial for tumor growth and perform a variety of roles in the development of tumors [30], which depends on their important role in the regulation of specific protein expression. *UCHL5* belongs to the ubiquitin C-terminal hydrolases (UCHs) family, which can remove Ub from the ubiquitin chain of the target protein, resulting in protein deubiquitination, and thereby reducing degradation [14–17]. In line with earlier research, we also found in this work that silencing *UCHL5* in HCC cells increased the degree of protein ubiquitination. Similarly, the target protein β-catenin is down-regulated, indicating that *UCHL5* is likely to inhibit protein degradation through the classical deubiquitination pathway. However, whether *UCHL5* directly binds to β-catenin or is related to the role of 26 S proteasome requires further experimental exploration. This regulatory relationship between *UCHL5* and β-catenin also exists in endometrial cancer. The presence of *UCHL5* up regulates and activates β-catenin, thereby promoting the proliferation of endometrial cancer [21, 31]. In this study, we confirmed that this regulatory mechanism also exists in HCC cells, and we first proposed that *UCHL5* regulates β-catenin in hepatocellular carcinoma by regulating its ubiquitination level in combination with β-catenin, which may provide a new potential direction for inhibiting β-catenin pathway.

The main protein of the Wnt/β-catenin pathway, β-catenin protein, plays a crucial role in the proliferation and spread of tumors by controlling a number of genes’ transcription [32]. Numerous studies have demonstrated that the Wnt/β-catenin pathway functions abnormally in HCC [33, 34]. In this work, we discovered that the amount of *UCHL5* expression regulated the activity of the Wnt/β-catenin pathway. Additionally, elevated Wnt/β-catenin pathway related proteins, including CyclinD1, c-Myc, Survivin, and VEGF, were also activated. Concerning these genes, the β-catenin inhibitor IWP-4 counteracted their overexpression. Additionally, the Wnt/β-catenin pathway promotes HCC cells to enhance glycolysis.

One of the major hallmarks of cancer is the unlimited proliferation and spread of tumor cells, which may encounter the energy needed for their proliferation and metastasis via abnormal glucose metabolism. This effect is called the “Warburg effect”, which is characterized by the transfer of energy production from oxidative phosphorylation to glycolysis [35, 36]. Enhanced cell glycolysis leads to the accumulation of its product lactic acid in the tumor microenvironment, this can increase tumor cell survival and assist tumor cells in evading the host’s immune surveillance. [37–40]. This metabolic abnormality affects the progression of HCC [41]. The Wnt/β-catenin pathway is a crucial mechanism for controlling metabolism, particularly glycolysis, in HCC, as demonstrated by earlier research [42]. This is in line with the findings of this investigation. Hepatocellular carcinoma-educated macrophages’ promotion of epithelial-mesenchymal transformation via Wnt2b/β-catenin /c-Myc signaling and reprogramming glycolysis [43]. In hepatocellular carcinoma cells, autophagy increases MCT1 expression and activates the Wnt/β-catenin signaling pathway, promoting metastasis and glycolysis [44]. Through blocking the Warburg Effect through the PPARγ-Dependent WNT/β-catenin /Pyruvate Dehydrogenase Kinase Isozyme 1 Axis, PPARγ Coactivator-1 Suppresses Metastasis of Hepatocellular Carcinoma [45]. Gankyrin stimulates glycolysis and glutaminolysis via upregulating c-Myc and activating β-catenin signaling to enhance tumorigenesis, metastasis, and treatment resistance in human HCC [46]. Our study’s findings demonstrate that altering *UCHL5* expression can control the mRNA expression levels of markers associated with metabolism, including GLUT1, PKM2, *HK2*, and *LDHA*, and further encourage the growth of glycolysis.

In this work, we discovered that *UCHL5* triggers Wnt/β-catenin to activate glycolysis. The final intuitive effect is that high *UCHL5* expression promotes glycolysis of HCC cells to provide energy for the proliferation and metastasis of HCC cells, this is unreported in earlier investigations. At the same time, we also found that the intracellular metabolite lactic acid is also increased. This finding provides some basis for further clarifying the molecular mechanism of HCC development, while targeting UCHL5 may be a potential predictive and therapeutic strategy for the clinical treatment of HCC. Unfortunately, we have not evaluated whether the metabolite lactic acid is discharged into the microenvironment and whether it reshapes the microenvironment through lactic acid, leading to cell metastasis, which requires us to further explore the detailed mechanism of *UCHL5*. Meanwhile, given the relatively high incidence of HCC, the clinical samples we collected were limited, and we need to further expand the sample size for more in-depth mechanistic and clinical studies.

## Conclusions

In summary, our study revealed that *UCHL5* is significantly expressed in hepatocellular carcinoma and speeds up tumor growth by encouraging hepatocellular carcinoma cell proliferation and metastasis. First, we discovered that *UCHL5* stimulates the Wnt/β-catenin pathway in tumor cells to enhance glycolysis. By interacting with β-catenin, *UCHL5* lowers its amount of ubiquitination, preventing β-catenin from being degraded and increasing its intracellular concentration. After activating the Wnt/β-catenin pathway, many genes including cell cycle detection points are activated, which eventually promotes the progression of HCC and has a poor prognosis. This opens new possibilities for clinical prediction and treatment of HCC.

### Electronic supplementary material

Below is the link to the electronic supplementary material.

Supplementary Material 1

Supplementary Material 2

## Data Availability

The datasets used and analyzed during the current study are available from the corresponding author upon reasonable request.
