# Supplementary figures and images for: UCHL5 promotes hepatocellular carcinoma progression by promoting glycolysis through activating Wnt/β-catenin pathway
Source: BMC Cancer. 2024 May 21;24:618. doi: 10.1186/s12885-023-11317-z (PMC11110341; doi:10.1186/s12885-023-11317-z)

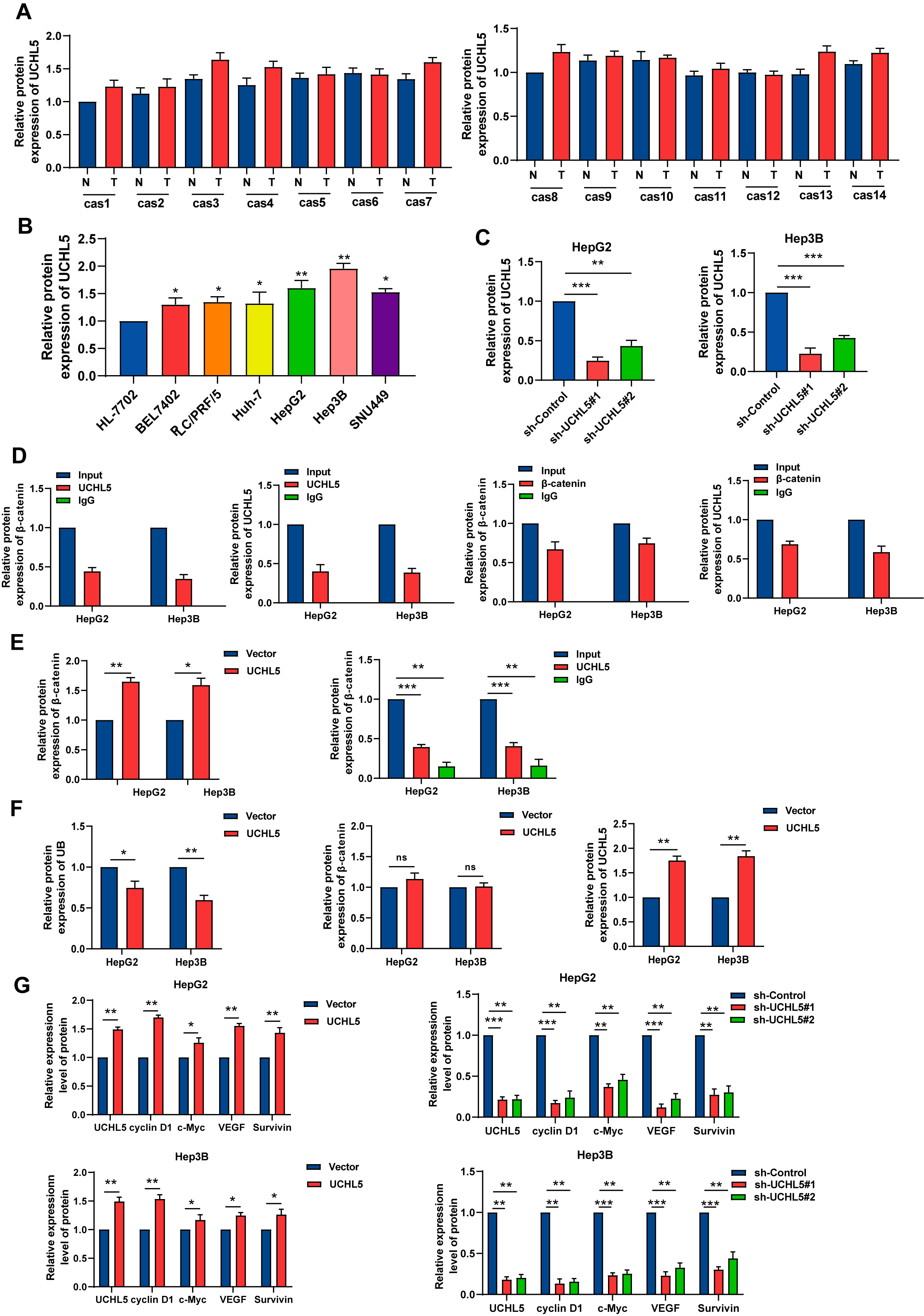

Supplement: Supplementary file 1 — Supplementary Material 1 [file 12885_2023_11317_MOESM1_ESM.png]
