# Supplementary material for: UCHL5 promotes hepatocellular carcinoma progression by promoting glycolysis through activating Wnt/β-catenin pathway
Source: BMC Cancer. 2024 May 21;24:618. doi: 10.1186/s12885-023-11317-z (PMC11110341; doi:10.1186/s12885-023-11317-z)
Supplement: Supplementary file 2 — Supplementary Material 2 [file 12885_2023_11317_MOESM2_ESM.docx]

Data 1

Data 2

Date 3
